# Rather sooner than later: Participatory change management associated with greater job satisfaction in healthcare

**DOI:** 10.1111/jan.15133

**Published:** 2022-01-12

**Authors:** Tuuli Turja

**Affiliations:** ^1^ Faculty of Social Sciences Tampere University Tampere Finland

In their article, Jakobsen et al. (2020) open a discussion about the importance of social capital in organizational changes in the specific context of the healthcare sector. This is considered as particularly timely research topic in a time when robots and other smart technologies are transforming many, also novel lines of work. Robotic devices are gradually reaching even the care work domain, which is consensually understood as a human‐centred and sensitive line of work (Turja & Parviainen, 2020). The renewal of service work underlines the need for modern era technological changes to acknowledge the importance of social capital and human well‐being at work rather than focusing solely on cost‐effectiveness and financial capital.

We can take robotization as an example of systematic organizational change that has swept through industrial manufacturing during the past 60 years. In industrial work, robotic investments have typically been separate from any co‐operation procedures between employers and employees. The introduction of robots has been considered mandatory and, for most, has made human labour more monotonous (Turja et al., in review). To promote psychological and social well‐being in technological and other organizational changes, one obvious direction is to include staff in planning such changes. By sharing decision‐making already in the planning stages of organizational changes, employers would be taking an important step toward social responsibility in the workplace.

This commentary lends support to the conclusion by Jakobsen et al. (2020), which states that participatory interventions may improve social capital in organizational changes. As our empirical findings will show, the mere sharing of knowledge about change before the implementation is associated with workplace social capital and feelings of respect as indicators of greater job satisfaction.

## 1 EMPIRICAL STUDY ON INCLUDING PERSONNEL IN ORGANIZATIONAL CHANGES

The objective of this commentary is to contribute to the discussion with empirical, self‐report data from Finnish healthcare professionals (*N* = 676). This subsample of healthcare sector professionals who reported their experiences of a workplace change was drawn from a larger sample of the Finnish Quality of Work Life Survey (QWLS) undertaken by Statistics Finland (2018) and yields national, representative interview data of Finnish salary‐earners (*N* = 4109).

Most of the 676 respondents in the subsample were female (88.4%) with an age range from 16 to 68 (mean = 44.97, *SD* 12.12). Almost half, 46.8%, of the healthcare sector workers had a college degree and 48.3% had a university degree.

When asked about the stage at which they were notified about organizational changes, 28.0% of respondents reported receiving information already in the planning phase, 49.1% receiving information somewhat before the change and 22.9% receiving information during or after the change.

The stage of being informed was analysed by nonparametric tests of independent samples, Kruskal–Wallis (*H*) because of the skewed distributions in the dependent variables. As the first finding, the earlier the stage at which the information about organizational change was received, the more satisfied the respondents were about their opportunities to generally exert influence in their workplaces (*H* = 38.85; *p* < .001). Those who were informed about the change only during or after the introduction of the change were prone to perceive their influence in the workplace as relatively poor.

Receiving information about organizational changes was analysed also in relation to perceived respect, collegial capital and satisfaction with management.

Perceived respect consisted of two items. (1) ‘Do you feel like a valued member in your work community’? With a response scale from 1 to 4 (*always… never*), and (2) ‘Are you satisfied with the respect you receive as a professional’? With a response scale from 1 to 4 (*extremely satisfied… not at all satisfied*). Higher values in the composite variable referred to lack of perceived respect (mean = 1.92, *SD* 0.65, *ρ* = 0.58). The reliability was estimated using Spearman–Brown split‐half coefficient (*ρ)* and because of the relatively low estimate of test score reliability (Rust & Golombok, 2014), the items of perceived respect were analysed also separately. The variation in perceived respect depended on receiving information about organizational changes (*H* = 31.64; *p* < .001). The earlier the stage at which the respondent reported receiving information about the organizational changes, the more respected they felt in their work and work community. Analysing the separate items, it was further confirmed that the earlier one perceived receiving information about changes, the more probable it was for them to feel like a valued member in the work community (*H* = 10.81; *p* < .005) and be satisfied with the respect they receive as professionals (*H* = 34.21; *p* < .001).

Collegial capital was measured with four statements in a Likert scale from 1 to 4: (1) ‘My coworkers encourage and support me when work feels difficult’, (2) ‘Work‐related problems and mistakes are openly discussed in our team’, (3) ‘I am generally satisfied with our team work’, (4) ‘I am satisfied with the social relations at the workplace’. Higher values in the composite variable referred to lower collegial capital (mean = 1.67, *SD* 0.58, *α* = 0.61). The reliability was estimated using Cronbach's alpha. Variation in collegial capital depended on receiving information about organizational changes (*H* = 12.83; *p* < .005). In a post hoc analysis, pairwise comparisons of the Dunn's test (with Bonferroni adjustments) indicated that the significant difference in perceived collegial capital was found between those who reported being informed about organizational changes already in the planning phase and those who reported being notified during or after the changes (*p* < .001).

Satisfaction with managers was constructed from five items concerning the respondent's immediate manager in the workplace. The responses were given on a Likert scale from 1 to 5: (1) My manager acknowledges my work, (2) My manager is inspiring, (3) My manager trusts the employees, (4) My manager shares responsibility in a meaningful way, (5) I am satisfied with my manager's style of leadership. Higher values in the composite variable referred to lower satisfaction (mean = 2.17, *SD* 0.93, *α* = 0.88). The reliability was estimated using Cronbach's alpha. Again, variation in satisfaction with management depended on receiving information about organizational changes (*H* = 69.24; *p* < .001). The earlier the respondents reported receiving information about organizational changes, the more satisfied they were with their managers.

## 2 SUMMARY

While shared decision‐making in organizational changes in healthcare work needs more advanced study designs, these preliminary results already lend support to the study by Jakobsen et al. (2020) and the importance of participatory methods in implementing changes in the workplace. Reorganizing work in cooperation with the staff not only promotes readiness for change among the employees but also manifests as greater social capital and feelings of respect after the change.

Participatory efforts concerning organizational changes seem to have temporal importance. Staff should be taken aboard rather sooner than later. From the perspective of job satisfaction, informing employees about forthcoming changes seems to be most beneficial at the earlier stages of the change. Those healthcare workers who received information about organizational changes in the planning stage of the change felt more respected, had stronger collegial capital, were more satisfied with having workplace influence and were more satisfied with their managers.

Involving employees in organizational changes at an early stage is one step in shared decision‐making in the healthcare sector. Healthcare organizations are typically hierarchical workplaces (Kuokkanen et al., 2007; Sellman, 2010) and introducing organizational changes to co‐operational procedures would perhaps be a small step for an organization, but a giant leap in renewing the management of healthcare work.

## CONFLICT OF INTEREST

None.
